# Meeting and exceeding dairy recommendations: effects of dairy consumption on nutrient intakes and risk of chronic disease

**DOI:** 10.1111/nure.12007

**Published:** 2013-01-30

**Authors:** Beth H Rice, Erin E Quann, Gregory D Miller

**Affiliations:** Dairy Research InstituteRosemont, Illinois, USA

**Keywords:** blood pressure, bone, cardiovascular disease, milk, type 2 diabetes

## Abstract

The 2010 *Dietary Guidelines for Americans* indicate the US population is experiencing an epidemic of overweight and obesity while maintaining a nutrient-poor, energy-dense diet associated with an increased risk of osteoarthritis, cardiovascular disease, and type 2 diabetes. To build upon the review of published research in the *Report of the Dietary Guidelines Advisory Committee on the Dietary Guidelines for Americans,* 2010, this article aims to review the scientific literature pertaining to the consumption of dairy foods and the effects of dairy consumption on nutrient intakes and chronic disease risk published between June 2010, when the report was released, and September 2011. PubMed was searched for articles using the following key words: dairy, milk, nutrient intake, bone health, body composition, cardiovascular disease, type 2 diabetes, and blood pressure. Evidence indicates that increasing dairy consumption to the recommended amount, i.e., three servings daily for individuals ≥9 years of age, helps close gaps between current nutrient intakes and recommendations. Consuming more than three servings of dairy per day leads to better nutrient status and improved bone health and is associated with lower blood pressure and reduced risk of cardiovascular disease and type 2 diabetes.

## Introduction

Nearly 70% of adults and one-third of children in the United States are overweight or obese.^1^,^2^ Despite being in a state of positive energy balance, Americans, on average, are not meeting recommended intakes for whole grains, vegetables, fruits, low-fat and fat-free dairy foods, seafood, and oils; they are also failing to meet recommended nutrient intakes for potassium, dietary fiber, calcium, and vitamin D, all of which were identified by the 2010 *Dietary Guidelines for Americans* (DGA) as nutrients for which intake is low enough to be of public health concern.^3^–^5^ In the United States, milk is the primary dietary source of three of these nutrients: calcium, vitamin D, and potassium.^6^

To help Americans meet nutrient recommendations, the 2010 DGA recommends 3 cups per day of fat-free or low-fat milk and milk products for persons 9 years of age and older, 2.5 cups per day for children 4–8 years of age, and 2 cups per day for children 2–3 years of age.^3^ Data from the National Health and Nutrition Examination Surveys (NHANES) indicate that meeting or exceeding current recommendations for dairy food intake can increase the dietary intake of several nutrients that are underconsumed in the United States, such as calcium, magnesium, and potassium.^7^

The 2010 DGA recognizes that, in addition to dairy foods being a source of essential nutrients, “moderate evidence shows dairy consumption is associated with improved bone health, especially in children and adolescents, and a reduced risk of cardiovascular disease and type 2 diabetes and with lower blood pressure in adults.”^3^ To build upon the review of published research in the Report of the Dietary Guidelines Advisory Committee (DGAC) that led to this conclusion, this article reviews the scientific literature pertaining to dairy food consumption and the effects of dairy consumption on nutrient intakes and risk of chronic disease published between June 2010, when the DGAC report was released, and September 2011.

## Dairy Consumption and Nutrient Intake

**Figure 1:**
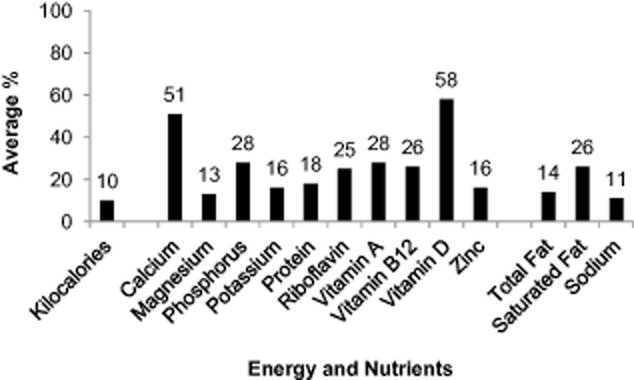
Percent energy and select nutrient contributions of dairy foods to the US diet from 1.8 average servings consumed per day Data obtained from day 1 of the 2003–2004 and 2005–2006 National Health and Nutrition Examination Surveys 24-hour dietary recall for individuals 2 years and older, excluding pregnant and lactating females (*n* = 16,822). Mixed dishes were disaggregated using the USDA Nutrient Database for Standard Reference food codes and linked to the appropriate food composition databases using the SR-Link file of the Food and Nutrient Database for Dietary Studies (FNDDS 2.0 and 3.0, based on SR 18 and SR 20, respectively), allowing for determination of the contribution of dairy foods used in mixed dishes to total nutrient intake.^9^

The dairy food group, which includes milk, cheese, and yogurt, contributes to the intake of many nutrients in the American diet, including more than 50% of total vitamin D and calcium and more than 25% of vitamin A, vitamin B_12_, and phosphorus, while contributing just 10% of total calorie intake (Figure 1).^8^–^10^ Dairy intake, however, is significantly below the levels recommended for most Americans,^8^ with individuals 2 years of age and older consuming 1.8 servings daily on average^9^ and only 15% of the population meeting recommendations for consumption of dairy foods.^11^ The DGA encourages increased consumption of fruits, vegetables, whole grains, lean protein sources (e.g., seafood, nuts, legumes), and low-fat and fat-free dairy foods to help individuals meet nutrient recommendations. A review of NHANES data from 2003–2004 indicated that three servings of dairy per day is the minimum amount necessary to ensure adequate intakes of calcium for Americans 9 years of age and older, but four servings or more per day may be necessary to ensure adequate intakes of magnesium and potassium.^7^ A recent study that utilized NHANES 2003–2006 data to examine the impact of adding one serving of dairy to current intakes supports this conclusion. The addition of a consumption-weighted composite of dairy foods (milk, cheese, and yogurt) increased average daily dairy intake to 2.8 servings and average calcium, magnesium, and potassium intakes to 1,245 mg, 301 mg, and 2,916 mg, respectively.^12^ Changes in additional dietary intakes are presented in Table 1. According to another analysis of NHANES data, from 2003 to 2006, more than one-third of Americans 2 years of age and older have usual intakes below the estimated average requirement (EAR) for vitamin A and calcium, nearly half have intakes below the EAR for magnesium, more than two-thirds have intakes below the EAR for vitamin D, and just 3% of the population have usual intakes of potassium greater than the adequate intake.^5^ Despite the naturally occurring amounts of these nutrients in food sources and the additional contribution from enriched foods and dietary supplements, shortfalls still exist.^5^

**Table 1:** Current dairy and nutrient intakes, based on NHANES 2003–2006 data (day 1, consumers aged 2 years and older, *n* = 16,882)^9^ and the dietary impact if one serving of dairy (consumption-weighted composite of milk, cheese, and yogurt) was added

Nutrient	Current intake (mean ± SE)^a^	One serving of dairy added (mean ± SE)^a^
Dairy (cup equivalent)	1.8 ± 0.0	2.8 ± 0.0**
Energy (kcal)	2,176 ± 16	2,319 ± 16**
Protein (g)	81.1 ± 0.8	90.3 ± 0.8**
Total fatty acids (g)	82.3 ± 0.8	89.1 ± 0.8**
Saturated fatty acids (g)	27.7 ± 0.3	31.9 ± 0.3**
Vitamin A, RAE (μg)	605 ± 10	721 ± 10**
Riboflavin (mg)	2.26 ± 0.03	2.64 ± 0.03**
Niacin (mg)	24.3 ± 0.3	24.6 ± 0.3
Vitamin B_12_ (μg)	5.34 ± 0.09	6.32 ± 0.09**
Vitamin D (μg)	4.96 ± 0.11	7.11 ± 0.1**
Calcium (mg)	944 ± 12	1,245 ± 12**
Phosphorus (mg)	1,329 ± 13	1,573 ± 13**
Magnesium (mg)	277 ± 3	301 ± 3**
Zinc (mg)	12.1 ± 0.1	13.4 ± 0.1**
Sodium (mg)	3,422 ± 28	3,634 ± 28**
Potassium (mg)	2,619 ± 25	2,916 ± 25**

** *P* < 0.01, significant difference between means before and after modeling diet change.

a Sample-weighted means and standard errors were estimated using SUDAAN® statistical software.

*Abbreviations*: RAE, retinol activity equivalents.

Adapted from Fulgoni et al. (2011).^12^

Several randomized clinical trials (RCTs) have demonstrated that consuming three or more servings of dairy foods per day has beneficial effects on nutrient intakes in adults.^13^–^16^ In middle-aged obese men and women fed a weight-maintenance diet for 6 months, the consumption of three or more servings of dairy foods per day resulted in significantly higher intakes of energy and macronutrients as well as of calcium and vitamin D compared with intakes of individuals who consumed one or fewer serving of dairy foods per day. For those who consumed higher amounts of dairy foods, the average calcium intake at the end of the study (women, 1,330 ± 39 mg/day; men, 1,448 ± 41 mg/day) was above the EAR (800 mg/day) for their age group, and vitamin D intakes doubled compared with baseline intake levels (women, 3.6 ± 0.5 to 7.1 ± 0.4 μg/day; men, 4.9 ± 0.8 to 9.7 ± 0.6 μg/day), which approached the EAR (10 μg/day) for their age group. For those who consumed lower amounts of dairy foods, the average calcium intake after 6 months was below the EAR (women, 587 ± 45; men, 624 ± 36 mg/day) for their age group, and vitamin D intakes did not improve from baseline and were well below the EAR (women, 4.2 ± 0.6 to 2.9 ± 0.4; men, 4.1 ± 0.7 to 3.8 ± 0.5 μg/day) for their age group.^13^ In another study in which total energy intake did not differ between groups, the consumption of four servings of dairy foods daily for 21 weeks by middle-aged obese adults resulted in significantly higher calcium intakes (1,200 ± 370 mg/day) compared with intakes in the group who consumed two servings of dairy foods per day (668 ± 273 mg/day).^15^ The group who consumed four servings of dairy foods per day had calcium intakes above the EAR for their age group, whereas the group who consumed two servings of dairy foods per day did not reach the EAR for calcium intake.^15^ Another RCT showed that, in groups of postmenopausal women between the ages of 55 and 65 years, matched for total energy and macronutrient consumption, consumption of three servings of low-fat calcium- and vitamin-D-fortified dairy products (2 servings of milk and 1 serving of yogurt) per day for 30 months resulted in no significant difference in intakes of total calories or macronutrients and significantly greater intakes of calcium, magnesium, vitamin D, and phosphorus (1,337 ± 500 mg/day, 329 ± 124 mg/day, 18.5 ± 1.4 μg/day, and 1,561 ± 675 mg/day, respectively) compared with intakes in a control group who did not consume three servings of fortified dairy products daily (564 ± 310 mg/day, 247 ± 104 mg/day, 0.8 ± 1.4 μg/day, and 1,133 mg/day, respectively).^16^ Similarly, in another study in which three servings of low-fat calcium- and vitamin-D-fortified dairy products were provided to postmenopausal women daily for 30 months, calcium, phosphorus, and vitamin D intakes were higher (1,183 ± 283 mg/day, 1,409 ± 627 mg/day, and 18.8 ± 1.3 μg/day, respectively) compared with intakes in the control group (671 ± 334 mg/day, 1,091 ± 484 mg/day, and 1.2 ± 0.6 μg/day, respectively).^14^ There were no significant differences in calorie, carbohydrate, or fat intakes, and protein intake was significantly higher in the dairy intervention group. Both of these studies reported intakes that increased from sub-EAR levels to levels that exceeded the EARs for calcium, vitamin D, and magnesium (1,000 mg/day, 10 μg/day, and 265 mg/day, respectively).^14^,^16^ These higher nutrient intakes were a significant improvement for this age group, which may be at risk for underconsuming calcium and vitamin D.^17^ The results of these studies indicate that exceeding daily dairy recommendations has beneficial effects on nutrient intakes.

## Dairy Consumption and Bone Health

Based on the review of the scientific literature by the DGAC, the 2010 DGA stated there was moderate evidence linking dairy consumption with improved bone health, especially in children and adolescents.^3^ Since the release of the DGAC report, several RCTs reinforced that dairy consumption also has benefits on bone health in adults, particularly in women (Table 2).^14^,^16^,^18^–^20^ In a 4-month trial in overweight premenopausal women who consumed a calorie-restricted diet, three or more daily servings of low-fat dairy foods (average daily calcium intakes above 1,300 mg) combined with resistance exercise increased lumbar spine bone mineral density compared with one or fewer servings of low-fat dairy foods per day combined with resistance exercise.^20^ In another study in healthy, young premenopausal women who followed a 12-week diet and resistance-exercise plan, four daily servings of fat-free milk added to participants' normal diets resulted in improved markers of bone turnover compared with an isoenergetic carbohydrate control.^19^ In the group that consumed four additional servings of fat-free milk per day, serum vitamin D (25-[OH]-D) increased and parathyroid hormone decreased more than in the group that did not consume any additional fat-free milk per day.^19^

**Table 2:** Effects of dairy intake on markers of bone health: summary of randomized clinical trials conducted between June 2010 and September 2011, using the search terms “dairy,” “milk,” “bone health,” and “body composition”

Reference	Characteristics of participants	Study objective	Dairy servings per day in experimental group	Results
Josse et al. (2010)^19^	Young healthy women (*n* = 10) and matched controls (*n* = 10), 23.2 ± 2.8 years of age	Determine the effects of 500 mL fat-free milk versus isoenergetic CHO control immediately and 1 h post exercise daily on body composition after 12 weeks	4	Milk group showed increased osteocalcin (*P* < 0.05), decreased carboxy-terminal collagen crosslinks C-telopeptides (*P* < 0.005), and no effect on bone-specific alkaline phosphatase compared with control group; serum 25-(OH)-D increased in milk group and control group (*P* < 0.05); serum 25-(OH)-D increased more in milk group than in control group (6.5 ± 1.1 versus 2.8 ± 1.3 nM, *P* < 0.05); PTH decreased in milk group only (−1.2 ± 0.2 pM, *P* < 0.01); control group and milk group gained lean mass (*P* < 0.01); milk group gained more lean mass than control group (1.9 ± 0.2 versus 1.1 ± 0.2 kg, *P* < 0.01); fat mass decreased in milk group only (−1.6 ± 0.4 kg, *P* < 0.01); isotonic strength increased in milk group only (*P* < 0.05); control group gained weight after training (0.86 ± 0.04 kg, *P* < 0.05)
Habibzadeh (2010)^18^	Obese (*n* = 9) and thin (*n* = 10) women and obese (*n* = 9) and thin (BMI < 20; *n* = 10) matched controls, 20–24 years of age	Assess the effect of 2 servings of milk/day, 3 times per week, on BMD for 2 months	2	BMD increased in hip and spine of obese and thin experimental groups by 4–7% (*P* < 0.05); body fat decreased in the thin experimental group (−8%, *P* < 0.05); lean body mass increased in the thin experimental group (3%, *P* < 0.001); serum calcium decreased in all groups except obese controls (3–5%, *P* < 0.05).
Thomas et al. (2010)^20^	Overweight women, 29−45 years of age (*n* = 29)	Examine lower-dairy calcium versus higher-dairy calcium diet, combined with resistance exercise, on body composition after 16 weeks	≥3	Lumbar spine BMD increased in the higher-dairy calcium group versus the lower-dairy calcium group (0.8% versus −1.5%, *P* < 0.05), higher-dairy calcium group consumed 1,312 ± 183 mg/day calcium, lower-dairy calcium group consumed 454 ± 143 mg/day calcium
Hinton et al. (2010)^13^	Obese subjects, 40.8 ± 0.6 years of age (*n* = 49 men, *n* = 64 women)	Determine if recommended dairy and calcium intake affect BMD and BMC after 24 weeks	≥3	Total body BMD and weight maintenance did not differ between recommended and low dairy groups; recommended dairy group had higher intake than low dairy group for protein (92 ± 2.0 versus 75 ± 3.0 g/day, *P* < 0.05), calcium (1,330 ± 39.0 versus 587 ± 45.0 mg/day, *P* < 0.05), and vitamin D (7.1 ± 0.4 versus 2.9 ± 0.4 μg/day, *P* < 0.05); serum 25-(OH)-D decreased in both groups during weight maintenance (*P* < 0.05)
Palacios et al. (2011)^15^	Puerto Rican obese adults, 21–50 years of age (*n* = 20 women, *n* = 5 men)	Determine if high-dairy diet to provide ∼1,300 mg/day of calcium or high-calcium diet to provide ∼1,300 mg/day calcium (∼700 mg/day from diet, ∼600 mg/day from supplements) alters body composition and serum lipids after 21 weeks	4	No differences in total BMC, total BMD, total body lean mass, weight, BMI, total body fat mass, total body fat, or trunk fat mass between groups. TAG levels were lower in high-dairy group for women only (18%, *P* < 0.05). FFQ data: high-dairy group consumed 1,337 ± 380 mg/day calcium, high-calcium group consumed 988 ± 250 mg/day calcium, control group consumed 463 ± 325 mg/day calcium, which was lower than the high-dairy and high-calcium groups (*P* < 0.05). Food record data: high-dairy group consumed 1,200 ± 370 mg/day calcium, high-calcium group consumed 1,171 ± 265 mg/day calcium, control group consumed 668 ± 273 mg/day calcium, which was lower than the high-dairy and high-calcium groups (*P* < 0.001)
Moschonis et al. (2010)^14^	Postmenopausal women in dietary group (*n* = 35) and matched controls (*n* = 31), 59–60 years of age	Examine effects of 1,200 mg calcium and 7.5 μg vitamin D through fortified dairy products for 12 months followed by 1,200 mg calcium and 22.5 μg vitamin D for an additional 18 months on BMD	3	Dietary intervention resulted in more favorable changes in dietary group versus control group for arm (0.033 versus −0.047 g/cm^2^, *P* < 0.01), total spine (0.118 versus 0.049 g/cm^2^, *P* < 0.001), and total body BMD (0.003 versus −0.03 g/cm^2^, *P* < 0.01), total fat intake from baseline (31.7 ± 7.4 versus 35.2 ± 6.6 KJ, *P* < 0.05), calcium intake from baseline (1,182.8 ± 283.4 versus 678.6 ± 275.1 mg/day, *P* < 0.001), phosphorus intake from baseline (1,409.2 ± 626.9 versus 993.5 ± 280.9 mg/day, *P* = 0.046), magnesium intake from baseline (314.9 ± 107.9 versus 195.4 ± 46.6 mg/day, *P* < 0.001), and vitamin D intake from baseline (18.8 ± 1.3 versus 0.76 ± 1.09 μg/day, *P* < 0.001)
Tenta et al. (2011)^16^	Postmenopausal women in dietary group (*n* = 20) and matched controls (*n* = 20), 55–65 years of age	Examine effects of 1,200 mg calcium and 7.5 μg vitamin D through fortified dairy products for 12 months followed by 1,200 mg calcium and 22.5 μg vitamin D for an additional18 months on bone metabolism and bone mass indices	3	Serum PTH was lower in the dietary group versus the control group (38.5 ± 19.8 versus 48.8 ± 20.9 pg/mL, *P* = 0.049), serum 25-(OH)-D was higher in the dietary group versus control group (27.2 ± 8.4 versus 15.3 ± 6.0, *P* < 0.001), and serum RANKL was lower in the dietary group versus control group (0.35 ± 0.22 versus 0.47 ± 0.30, *P* = 0.005). Protein intake was higher in the dietary group versus control group (14.6 ± 3.9 versus 12.2 ± 4.4% kcal, *P* = 0.02), calcium intake was higher in the dietary group versus control group (1,336.6 ± 500 versus 563.6 ± 309.5 mg/day, *P* < 0.001), phosphorus intake was higher in the dietary group versus control group (1,561.0 ± 674.6 versus 1,132.7 ± 539.1 mg/day, *P* = 0.01), magnesium intake was higher in the dietary group versus control group (329 ± 120.4 versus 247.3 ± 104.2 mg/day, *P* < 0.001), and vitamin D intake was higher in the dietary group versus control group (18.47 ± 1.42 versus 0.77 ± 1.38 μg/day, *P* < 0.001)
Campbell & Tang (2010)^51^	Study 1: 28 postmenopausal women, 51–60 years of age. Study 2: 54 postmenopausal women, 51–60 years of age	Examine effects of higher-protein diets on bone. Study 1: subjects consumed energy-restricted diet: lacto-ovo diet with 18% energy from protein (*n* = 15 subjects) versus omnivorous diet with 30% energy from protein (*n* = 13 subjects) for 12 weeks. Study 2: subjects consumed habitual diet (control, *n* = 11 subjects) versus energy-restricted diet with 16% energy from nonmeat protein sources (*n* = 14 subjects) or 26% energy from chicken (*n* = 15) or beef (*n* = 14) protein sources for 9 weeks	<2	Study 1: BMD decreased with weight loss in higher-protein diet only (−0.0167 ± 0.004 g/cm^2^, *P* < 0.05). Study 2: BMD values decreased in chicken (−1.1%, *P* < 0.05) and beef (−1.4%, *P* < 0.05) groups compared with baseline values

*Abbreviations*: BMD, bone mineral density; BMC, bone mineral content; BMI, body mass index; CHO, carbohydrate; FFQ, food frequency questionnaire; PTH, parathyroid hormone; RANKL, receptor activator of nuclear factor-kappaB ligand; TAG, triglyceride; 25-(OH)-D, 25 hydroxy-vitamin D.

Serum 25-(OH)-D levels above 80 nM/L have been related to improved bone health.^17^ Participants who consumed the four additional servings of fat-free milk each day had serum 25-(OH)-D levels that increased to between 48.2 nM/L and 79.2 nM/L; thus some were still vitamin D insufficient (50–80 nM/L) or deficient (<50 nM/L).^17^,^19^ The beneficial effects on markers of bone health that resulted from consuming four additional servings of fat-free milk per day may have been due to the approximately 1,200 mg of added calcium contributed by milk to the diet, which allowed serum calcium to be maintained within the recommended circulating range.^19^ Greater gains in lean muscle mass and a decrease in fat mass compared with the control group were also observed, showing a beneficial effect of four daily servings of milk on total body composition during weight loss.^19^ In a trial that examined the effect of milk consumption on body composition in lean and obese premenopausal females, an average of just two added servings of reduced-fat milk three days a week to the normal diet of both lean and obese participants for 8 weeks resulted in increased bone mineral density of the hip and spine compared with that in controls who did not consume any additional dairy foods.^18^ Additionally, lean body mass increased and percent body fat decreased in the lean milk-consuming group.^18^ The results of these studies indicate that exceeding daily dairy recommendations may have beneficial effects on markers of bone health and body composition in female adults.

In a study of obese men and premenopausal women who participated in a 12-week weight-loss intervention followed by a 24-week weight-maintenance period, total body bone mineral content was increased from baseline in women who consumed ≥3 servings of dairy per day (recommended) as well as in those who consumed ≤1 serving of dairy per day (low consumption).^13^ Despite increases in total body bone mineral content, no changes in total body bone mineral density were detected.^13^ In the men, both low dairy consumption and recommended dairy consumption resulted in increased total body bone mineral density over the course of the intervention. Total body bone mineral content, however, decreased in the men over time.^13^ Estrogen is known to inhibit bone resorption,^21^ perhaps explaining why differences in treatment effects on bone health were observed between the women and the men. Why differences in total body bone mineral content were not reflected in total body bone mineral density cannot be explained. In another trial, Palacios et al.^15^ reported that Puerto Rican obese men and women who consumed four servings of low-fat milk, cheese, and yogurt daily for 21 weeks had no differences in total body bone mineral content or density from baseline or compared with those who consumed 1,200–1,300 mg of calcium per day from a dietary supplement or those who consumed less-than-recommended amounts of dairy foods and no calcium supplement (<700 mg calcium/day). Mixed results from these two trials that studied dairy intake and bone health in obese subjects indicate that obesity, in addition to gender, may have a considerable effect on bone health regardless of dairy and micronutrient intakes. This may be because, in obese individuals, compounds vital to bone health such as vitamin D are incorporated into adipose tissue and do not contribute to serum 25-(OH)-D concentrations.^22^ Whereas trials in obese individuals have produced inconsistent results, RCTs in normal-weight individuals show a beneficial effect of dairy consumption on bone health.

## Dairy Consumption and Cardiovascular Disease

Dairy foods provide bioavailable calcium and have been associated with beneficial effects on risk of cardiovascular disease.^8^ Two meta-analyses that aimed to investigate the effects of calcium supplementation on cardiovascular events indicated that nondietary calcium supplementation may increase the risk of cardiovascular events in women over 40 years of age.^23^,^24^ The DGAC report indicated calcium naturally occurring in foods is the recommended source.^8^

Based on the DGAC's review of the scientific literature, which presented evidence from two systematic reviews and meta-analyses and one case-controlled study,^8^ the 2010 DGA stated, “Moderate evidence also indicates that intake of milk and milk products is associated with a reduced risk of cardiovascular disease and type 2 diabetes and with lower blood pressure in adults.”^3^ The 2010 DGA recommends the consumption of low-fat and fat-free milk and milk products (milk, yogurt, and cheese) as a means to obtaining the same nutrients that are available in full-fat varieties but with a reduced amount of calories and saturated fat.^3^ While dairy foods can contribute to saturated fat intake, the research summarized in the 2010 DGAC report indicates consumption of milk products may not have a predictable effect on serum lipids; it may not raise total cholesterol and may favorably impact high-density lipoprotein cholesterol.

Since the release of the 2010 DGAC report, several epidemiological studies have examined the associations between dairy consumption and cardiovascular disease endpoints, such as coronary heart disease and stroke (Table 3).^25^–^31^ A meta-analysis of prospective cohort studies examining the associations of milk and total dairy intakes with the risk of cardiovascular diseases in over 600,000 participants in the United States, Europe, and Japan found that milk intake was associated with a reduced risk of overall cardiovascular diseases (dose response of 6% reduced risk for each 200 mL/day).^30^ Mean intake among participants was one serving of dairy foods per day.^30^ Notably, when analyzed according to fat content, no association between high-fat, low-fat, or combined total fat content of dairy foods and coronary heart disease was detected, indicating a neutral effect of milk fat on risk of coronary heart disease.^30^

**Table 3:** Effects of dairy intake on risk factors for cardiovascular disease: summary of epidemiological studies conducted between June 2010 and September 2011, using the search terms “dairy,” “milk,” and “cardiovascular disease

Reference	Characteristics of participants	Study design and objective	Mean servings of dairy per day	Results
Soedamah-Muthu et al. (2011)^30^	Adults from the USA, Japan, and Europe, 56 ± 13 years of age (*n* = 611,430)	Meta-analysis of 17 prospective cohort studies to examine the associations of milk, total dairy, and high- and low-fat dairy intakes with the risk of CVD and total mortality over a mean follow-up of 14 ± 6 years	<2	Modest inverse association between milk intake and risk of overall CVD (4 studies: RR, 0.94 per 200 mL/day; 95%CI, 0.89–0.99); milk intake was not associated with risk of CHD (6 studies: RR, 1.00; 95%CI, 0.96–1.04), stroke (6 studies: RR, 0.87; 95%CI, 0.72–1.05), or total mortality (8 studies: RR per 200 mL/day, 0.99; 95%CI, 0.95–1.03); no association between total, total high-fat, or total low-fat dairy products (200 g/day) and CHD
Bernstein et al. (2010)^26^	American adult women, 30–55 years of age (*n* = 84,136)	Prospective study to examine the relationship between foods that are major dietary protein sources and incident CHD	<1–3	Low-fat and high-fat dairy foods substituted for red meat were associated with a decreased risk of CHD (*P* < 0.05) and, when substituted for fish, were associated with an increased risk of CHD (*P* < 0.05); a serving of high-fat dairy was associated with an increased hazard ratio for CHD (1.03; 95%CI, 1.00–1.06, *P* < 0.05)
Panagiotakos et al. (2010)^52^	Greek adults, 18–89 years of age (*n* = 1,514 men and 1,528 women)	Cross-sectional study to investigate the association between consumption of dairy products and levels of various inflammatory markers among adults with no evidence of CVD or other chronic diseases	1–2	1.5–2 servings of dairy/day associated with lower plasma total cholesterol, TAG, and hypercholesterolemia versus <1 serving/day (*P* < 0.05), regardless of full-fat or low-fat dairy (*P* > 0.76); 2 servings/day versus <1 serving/day associated with lower CRP (1.61 ± 1.8 versus 2.26 ± 1.6 mg/L, *P* = 0.32), IL-6 (1.34 ± 0.32 versus 1.48 ± 0.37 ng/mL, *P* = 0.001), and TNF-α (5.58 ± 2.8 versus 1.48 ± 0.37 mg/dL, *P* < 0.001); 1 additional serving of full-fat dairy/week associated with decreased IL-6 (β = −0.071 ± 0.05 mg/L, *P* = 0.02) and TNF-α (β = −0.047 ± 0.02 mg/dL, *P* = 0.02); 1 additional serving of low-fat dairy/week associated with decreased CRP (β = −0.071 ± 0.02 mg/L, *P* = 0.02), IL-6 (β = −0.066 ± 0.01 ng/mL, *P* = 0.03). and TNF-α (β = −0.040 ± 0.01 mg/dL, *P* = 0.01)
Goldbohm et al. (2011)^28^	Dutch adults, 55–69 years of age (*n* = 120,852)	Prospective study to investigate the association between dairy product consumption and the risk of death from all causes, IHD, and stroke	2	Butter and dairy fat intake increased risk of all-cause and IHD mortality in women only (Rate Ratio_mortality,_ 1.04 per 10 g/day; 95%CI, 1.01–1.06 per 10 g/day, *P* < 0.05); fermented full-fat milk was inversely associated with all-cause mortality
Aslibekyan et al. (2012)^25^	Costa Rican adults, 58–59 years of age (*n* = 3,360)	Case-control study to evaluate the association between dairy intake as measured by FFQ and adipose tissue content of C 15:0 and C 17:0 fatty acids with risk of nonfatal MI	1	No association between dairy consumption and risk of nonfatal MI
Bonthuis et al. (2010)^27^	Australian adults, 25–78 years of age (*n* = 1,529)	Prospective study to investigate the relationship between intake of dairy products or related nutrients and mortality due to CVD, cancer, and all causes over a follow-up period of 16 years	<1–2.5	Higher intake of full-fat dairy foods associated with decreased hazard ratios of CVD mortality (1.5 servings/day = 0.31; 95%CI, 0.12–0.79 versus <1 serving/day = 0.73; 95%CI, 0.35–1.54; *P* = 0.04)
Warensjö et al. (2010)^31^	Swedish adults, 50–60 years of age (*n* = 444 cases and 556 controls)	Case-control study to investigate the association between dairy intake as measured by adipose tissue content of C 15:0 and C 17:0 fatty acids and their sum with risk of first MI	1–2	Higher intake of dairy as indicated by biomarkers of milk fat was inversely associated with first MI (OR, 0.74; 95%CI, 0.58–0.94 in women); quartiles of reported intake of cheese were inversely related to first MI in men and women (*P* < 0.05); quartiles of reported intake of fermented milk products were inversely related to first MI in men (*P* < 0.05)
Esmaillzadeh & Azadbakht (2010)^53^	Tehrani women, 40–60 years of age (*n* = 486)	Cross-sectional study to assess the association between dairy consumption and circulating levels of inflammatory markers	<0.5	Low-fat dairy consumption was inversely associated with sVCAM-1 (β = −0.03, *P* < 0.05); high-fat dairy consumption was positively associated with serum amyloid A (β = 0.08, *P* < 0.05) and sVCAM-1 (β = 0.05, *P* < 0.05); and there was no association between overall dairy consumption and circulating markers of inflammation
Ivey et al. (2011)^29^	Australian women, >70 years of age (*n* = 1,080)	Prospective study to assess the association between dairy consumption and CCA-IMT after a period of 3 years	<0.5–1.2	Total dairy product, milk, and cheese consumption was not associated with CCA-IMT (*P* < 0.05); yogurt consumption was inversely associated with CCA-IMT (β = −0.075, *P* = 0.015); participants who consumed >0.5 servings of yogurt per day had lower CCA-IMT versus those who consumed <0.5 serving of yogurt per day (−0.023 mm, *P* = 0.003)

*Abbreviations*: C, carbon; CCA-IMT, common carotid artery intima-media thickness; CRP, C-reactive protein; CVD, cardiovascular disease; CI, confidence interval; CHD, coronary heart disease; FFQ, food frequency questionnaire; IHD, ischemic heart disease; IL-6, interleukin-6; MI, myocardial infarction; RR, relative risk; sVCAM-1, soluble vascular cell adhesion molecule; TAG, triglyceride; TNF-α, tumor necrosis factor-α.

In a prospective study among adult women in the United States, substituting dairy foods for fish was associated with increased risk of coronary heart disease, and consumption of high-fat dairy foods was associated with an increased risk of coronary heart disease.^23^ Consumption of low-fat and high-fat dairy foods, however, when compared with consumption of red meat, was associated with a substantially lower risk of coronary heart disease.^26^ The highest median dairy food intake was two servings daily.^26^ In another prospective investigation among Dutch adults who consumed on average three servings of dairy foods per day, fermented full-fat milk was inversely associated with all-cause mortality, but dairy fat was associated with an increased risk of all-cause and ischemic heart disease mortality among women only.^28^ In a case-control study of over 3,000 Costa Rican adults, however, no association between dairy fat and risk of myocardial infarction was detected.^25^ In another investigation of over 1,500 Australian adults followed for 16 years, higher intake of higher-fat dairy foods was associated with decreased risk of cardiovascular mortality; 69% less risk was associated with consuming about 1.5 servings of higher-fat dairy foods per day versus 27% less risk associated with consuming less than 0.5 serving of higher-fat dairy foods per day.^27^ In over 400 Swedish adults who consumed one to two servings of dairy foods daily, higher intake of dairy foods, as measured by biomarkers of milk fat in adipose tissue, was associated with 26% less likelihood of first myocardial infarction.^31^ The men and women studied consumed both low- and high-fat dairy products such as milk, fermented milk, cheese, and cream.^31^ In postmenopausal women in Australia, consumption of milk, cheese, and total dairy product (mean intake of 2.3 servings per day) was not associated with coronary heart disease as measured by common carotid artery intima-media thickness.^29^ Yogurt consumption was inversely associated with coronary heart disease, and in those women who consumed more than one-half serving of yogurt daily, arterial thickness was less than in those who consumed less than one-half serving daily.^29^ The results of these epidemiological studies indicate that low-fat dairy foods are associated with decreased risk of coronary heart disease. Whereas some studies pointed to detrimental effects, the majority indicated that full-fat dairy foods may have beneficial effects on risk of coronary heart disease. Research on the effects of milk fat on risk of coronary heart disease is ongoing.

Since the release of the DGAC report, four RCTs have examined the effects of dairy consumption on biomarkers associated with risk of cardiovascular disease (Table 4).^32^–^35^ In addition to traditional lipid screenings, biomarkers of inflammation have emerged as equally if not more valuable indicators of coronary heart disease risk.^36^ In overweight and obese subjects, a weight-maintenance diet including three daily servings of low-fat dairy smoothies that provided a total of 1,050 mg of calcium per day was shown to reduce biomarkers of oxidative stress and chronic inflammation within 28 days compared with a soy-based placebo smoothie that provided 500–600 mg of calcium per day.^35^ The dairy intervention also lowered serum low-density lipoprotein cholesterol and had no adverse effects on blood pressure or serum total cholesterol, high-density lipoprotein cholesterol, or triglycerides.^35^ Notably, in another study in postmenopausal women, when three servings of reduced-fat (2% fat) cow's milk was compared with a vanilla soy beverage containing comparable amounts of macronutrients and calcium for 1 month, no differences in serum lipids between groups, or from baseline, were detected.^32^ In middle-aged overweight and obese adults, it was demonstrated that daily consumption of three servings of low-fat milk and yogurt had beneficial effects on several biomarkers of inflammation.^34^ Another intervention designed to test the differences between effects of high-fat and low-fat dairy foods on biomarkers of inflammation following a single dairy meal showed that high-fat dairy foods such as butter, cheese, cream, and yogurt (45 g of fat per single-meal intervention) reduced several biomarkers of inflammation as well as levels of both low-density and high-density lipoprotein cholesterol.^33^

**Table 4:** Effects of dairy intake on biomarkers for cardiovascular disease risk: summary of randomized clinical trials conducted between June 2010 and September 2011, using search terms “dairy,” “milk,” and “cardiovascular disease

Reference	Characteristics of participants	Study objective	Dairy servings/day in experimental group	Results
Zemel et al. (2010)^35^	Obese and overweight subjects (*n* = 20; 14 men, 6 women), 31 ± 10.3 years of age	Examine the effects of a soy-based placebo weight-maintenance diet (500–600 mg calcium/day) versus a dairy diet (1,200–1,400 mg calcium/day) for 28 days on body composition, markers of oxidative and inflammatory stress, blood pressure, and other biochemical variables	3	Dairy diet reduced markers of oxidative stress (malondialdehyde, 22%, *P* < 0.05; 8-isoprostane F2α, 12%, *P* < 0.02) compared with soy (no effect); dairy decreased circulating TNF-α, IL-6, and CRP compared with soy (*P* < 0.05); dairy increased adiponectin compared with soy (*P* < 0.05); dairy decreased LDL cholesterol compared with soy (*P* < 0.05); treatments had no effect on blood pressure, total cholesterol, HDL cholesterol, or TAG
Nestel et al. (2012)^33^	Overweight subjects in single-meal intervention (*n* = 12) and overweight subjects in 4-week intervention (*n* = 12), 44–69 years of age	Examine the effect of full-fat dairy meals on inflammatory biomarkers of CVD risk after a single meal and after a 4-week intervention, measured against a fat-free milk control	Approximately 2	Single-meal effects: following each full-fat meal, plasma TAG increased (22%, butter; 20%, cheese; 40%, cream; 48%, yogurt; *P* < 0.05), LDL cholesterol decreased (−6 to −9%; *P* < 0.05), HDL cholesterol decreased after butter (−3%), cream (−6%), and yogurt (−7%), *P* < 0.05, plasma glucose decreased after butter (−10%) and cheese (−23%), *P* < 0.05; changes in inflammatory biomarkers were not different between groups; within groups, MCP-1α, MIP-1α, and sVCAM-1 were decreased in the cream group (*P* < 0.05), IL-6 was decreased in the butter, cream, and control groups (*P* < 0.05), IL-1β was decreased in the butter, cream, and control groups (*P* < 0.05), TNF-α was decreased in the butter, cream, and control groups (*P* < 0.05), and high-sensitivity CRP was decreased in the butter and cream groups (*P* < 0.05). There was no difference between the different fat groups after the 4-week intervention
Van Meijl & Mensink (2010)^34^	Overweight and obese adults, 50 years of age (*n* = 35)	Investigate the effects of low-fat dairy consumption (milk and yogurt) on inflammatory markers and adhesion molecules after an 8-week period	>2.5	Low-fat dairy consumption decreased the plasma TNF-α to TNF-α receptor-1 ratio (*P* = 0.015) but had no effect on other markers of chronic inflammation and endothelial function
Beavers et al. (2010)^32^	Postmenopausal women, 40–60 years of age (*n* = 32)	Examine the effect of reduced-fat cow's milk (4.5 g per serving) versus vanilla soy beverage (4 g of fat per serving) on plasma cholesterol concentrations after a period of 4 weeks	3	There were no differences in TAG, LDL cholesterol, or HDL cholesterol between cow's milk and soy beverage groups or from baseline in either group after the intervention

*Abbreviations*: CRP, C-reactive protein; CVD, cardiovascular disease; HDL, high-density lipoprotein; IL-6, interleukin-6; IL-1β, interleukin-1β; LDL, low-density lipoprotein; MIP-1α, macrophage inflammatory protein; MCP-1α, monocyte chemoattractant protein-1 α; sVCAM-1, soluble vascular cell adhesion molecule; TAG, triglyceride; TNF-α, tumor necrosis factor-α.

Collectively, these studies indicate an inverse association between consumption of dairy foods and coronary heart disease, adding to the evidence that led the DGAC to conclude that “bioactive components that alter serum lipid levels may be contained in milk fat.”^8^ Additionally, controlled trials that examined differences in coronary heart disease risk based on the fat level of dairy foods showed that the consumption of milk fat does not detrimentally – and may beneficially – affect biomarkers of coronary heart disease.

## Dairy Consumption and Type 2 Diabetes

The review of the scientific literature by the DGAC presented findings from a meta-analysis of four prospective studies and found the relative risk of type 2 diabetes was estimated to be 10% lower in people with higher milk intake than in those with lower consumption.^8^ Based on this finding, the 2010 DGA stated that moderate evidence indicates the intake of dairy foods is associated with a reduced risk of type 2 diabetes.^3^ Several epidemiological studies designed to assess the relationship between dairy consumption and type 2 diabetes have been published since the release of the 2010 DGAC report and all have reported an inverse association between the intake of milk and milk products and the risk of type 2 diabetes (Table 5).^37^–^40^ A prospective study that assessed how dairy consumption by adolescents was associated with the incidence of type 2 diabetes in adulthood found that adults who reported consistently consuming two servings of dairy per day during adolescence had a 43% less risk of developing type 2 diabetes in adulthood when compared with adults who reported consuming one-half serving or less of dairy per day during adolescence.^38^ Consumption of both low-fat and full-fat dairy foods in adolescence was inversely associated with type 2 diabetes in adulthood.^38^ A prospective study of older adults found that circulating *trans*-9 C16:1, a biomarker indicative of dairy fat consumption, was associated with a 62% lower incidence of type 2 diabetes in adults who consumed at least one serving compared to those who consumed less than one serving of reduced-fat (2% milk fat) and/or full-fat dairy foods daily.^39^ Another study conducted in French adults found that those who consumed more than three servings of milk or yogurt daily had a 15% lower risk of incident metabolic syndrome and/or type 2 diabetes compared with those who consumed less than one serving of milk or yogurt daily.^37^ Cheese consumption had no effect on the incidence of metabolic syndrome and/or type 2 diabetes.^37^ In a meta-analysis of over 300,000 American, Japanese, and Chinese middle-aged participants, higher dairy consumption (>3 servings/day versus <1 serving/day) was associated with a 15% reduced risk of type 2 diabetes.^40^ A dose-response analysis showed that the risk of type 2 diabetes could be reduced by 5% for each additional serving of total dairy products and by 10% for each additional serving of low-fat dairy products consumed.^40^ Findings from these epidemiological investigations indicate milk and milk products, regardless of fat level, are associated with a reduced risk of type 2 diabetes. Furthermore, exceeding current recommendations of three servings daily may further reduce risk.

**Table 5:** Effects of dairy intake on risk of type 2 diabetes: summary of studies conducted between June 2010 and September 2011, using search terms “dairy,” “milk,” and “type 2 diabetes”

Reference	Characteristics of participants	Study design and objective	Mean servings of dairy per day	Results
Epidemiological studies				
Malik et al. (2011)^38^	Women, 34–53 years of age (*n* = 37,038)	Prospective study to assess the relationship between dairy product consumption as reported through a diet recall FFQ and incidence of T2D in adulthood	0.5–2	Two servings of dairy per day associated with 27% lower risk of T2D (RR, 0.73; 95%CI, 0.54–0.97; *P* = 0.02) and was attenuated with adjustment for adult dairy product consumption. Multivariate analysis of adolescent and adult consumption showed consistently higher intakes of dairy were associated with the lowest risk of T2D (RR, 0.57; 95%CI, 0.39–0.82; *P* < 0.05)
Tong et al. (2011)^40^	American, Japanese, and Chinese adults (>23,000 cases), 39–57 years of age (*n* > 330,000)	Meta-analysis to elucidate the association between consumption of dairy products and T2D	0–≥3	Dairy consumption inversely associated with T2D (combined RR, 0.86; 95%CI, 0.79–0.92). Individual dairy foods were inversely or not associated with T2D: low-fat dairy (combined RR, 0.82; 95%CI, 0.74–0.90), high-fat dairy (combined RR, 1.00; 95%CI, 0.89–1.10), whole milk (combined RR, 0.95; 95%CI, 0.86–1.05), yogurt (combined RR, 0.83; 95%CI 0,74–0.93). Dose-response analysis showed T2D risk could be reduced 5% for total dairy products and 10% for low-fat dairy products
Fumeron et al. (2011)^37^	French adults, 30–65 years of age (*n* = 3,435)	Prospective study to assess the influence of milk and dairy products (not including cheese) versus cheese on incident MetS and impaired fasting glycemia and/or T2D after 9 years of follow-up	0.5–>1	Milk and dairy products (not including cheese) inversely associated with incident MetS and impaired fasting glycemia and/or T2D (OR, 0.85; 95%CI, 0.76–0.94; *P* = 0.001). Cheese not associated with incident MetS and impaired fasting glycemia and/or T2D (*P* > 0.05)
Mozaffarian et al. (2010)^39^	Adults, 75 years of age (*n* = 3,736)	Prospective cohort study to investigate whether circulating *trans*-9 C16:1 (a biomarker of ruminant milk and meat intake) is independently related to lower metabolic risk and incident T2D	1–2	Whole-fat dairy consumption (whole milk, 2% milk, cheese, butter, and ice cream) most strongly associated with higher *trans*-9 C16:1. *trans*-9 C16:1 associated with higher HDL cholesterol (1.9%, *P* = 0.04), lower TAG (−19%, *P* < 0.001), lower total cholesterol to HDL cholesterol ratio (−4.7%, *P* < 0.001), lower CRP (−13.8%, *P* = 0.05), lower insulin resistance (−16.7%, *P* < 0.001), and lower incidence of T2D (HR, 0.38; 95%CI, 0.24–0.62; *P* < 0.001)
Clinical trials				
Nikooyeh et al. (2011)^41^	Diabetic Iranian adults, 30–60 years of age (*n* = 90)	Examine the effects of 200 mL plain yogurt drink (150 mg Ca), vitamin-D-fortified yogurt drink (500 IU vitamin D and 150 mg Ca), and vitamin D + Ca-fortified yogurt drink (500 IU vitamin D and 250 mg Ca) twice daily for 12 weeks on fasting serum glucose, glycated hemoglobin, HOMA–IR, serum lipid profile, and percent fat mass	2	Vitamin-D-fortified and vitamin D + Ca-fortified yogurt drink decreased fasting serum glucose (*P* = 0.015), glycated hemoglobin (*P* < 0.001), HOMA–IR (*P* ≤ 0.001), waist circumference (*P* < 0.001), and BMI (*P* ≤ 0.005) compared with the plain yogurt drink
Stancliffe et al. (2011)^42^	Overweight and obese adults, 37 ± 9.9 years of age (*n* = 40)	Determine the early (7-day) and sustained (4- and 12-week) effects of higher-dairy (3.5 dairy servings/day) and lower-dairy (<0.5 serving/day) diets on oxidative and inflammatory biomarkers in subjects with MetS	<0.5–3.5	Higher-dairy diet decreased malondialdehyde and oxidized LDL cholesterol at 7 days (*P* < 0.01), suppressed TNF-α (*P* < 0.05), IL-6 (*P* < 0.02), and MCP-1 (*P* < 0.05), and increased adiponectin (*P* < 0.01) from baseline, whereas lower-dairy diet had no effect on oxidative or inflammatory markers

*Abbreviations*: BMI, body mass index; C, carbon; Ca, calcium; CRP, C-reactive protein; CI, confidence interval; FFQ, food frequency questionnaire; HDL, high-density lipoprotein; HOMA–IR, homeostasis model of assessment–insulin resistance; HR, hazard ratio; IL-6, interleukin-6; IU, international units; LDL, low-density lipoprotein; MCP-1, monocyte chemoattractant protein 1; MetS, metabolic syndrome; OR, odds ratio; RR, relative risk; TAG, triglyceride; T2D, type 2 diabetes; TNF-α, tumor necrosis factor-α.

Since the release of the 2010 DGAC report, two RCTs have investigated the effects of dairy consumption on risk factors associated with type 2 diabetes.^41^,^42^ It was demonstrated in middle-aged overweight and obese individuals that consumption of three and one-half servings of dairy per day, with two of the three being milk and/or yogurt (fat level not reported), resulted in increased insulin sensitivity and decreased plasma insulin within 1 week of consuming dairy foods, changes that were sustained throughout the 12-week duration of the study.^42^ Additionally, beneficial effects on several lipid and inflammatory markers of metabolic health were observed when comparing these individuals with those who consumed less than one-half serving of dairy daily.^42^ In middle-aged subjects with type 2 diabetes, vitamin-D- and calcium-fortified yogurt drinks consumed twice daily for 3 months led to decreases in fasting serum glucose, glycated hemoglobin, and insulin resistance measured by the Homeostasis Model of Assessment–Insulin Resistance, which are indicators of glycemic status.^41^

## Dairy Consumption and Blood Pressure

The 2010 DGA stated there was moderate evidence that dairy consumption is associated with lower blood pressure in adults.^3^ Dietary patterns that incorporate at least three servings of dairy daily, such as the Dietary Approaches to Stop Hypertension eating plan, which includes low-fat milk, cheese, and yogurt and one serving of full-fat cheese each day, have been successful in reducing blood pressure.^8^ The DGAC, however, noted that an independent relationship between dairy intake and blood pressure is complicated to evaluate due to varying types of dairy products consumed in research trials, confounding effects of calcium intake from other food sources, and the beneficial effect of weight loss on blood pressure.^8^ A systematic review and meta-analysis of studies evaluating the association between dairy intake and blood pressure in approximately 45,000 adults found that consumption of dairy foods was associated with a 13% reduced risk of elevated blood pressure.^43^ Separation of high-fat and low-fat dairy foods revealed an inverse association between only low-fat dairy foods and elevated blood pressure, with no effect from high-fat dairy foods or cheese.^43^

Since the release of the 2010 DGAC report, four RCTs have investigated the effects of dairy foods on blood pressure and components of blood pressure control (Table 6).^42^,^44^–^46^ While other RCTs have tested the effects of specific dairy ingredients on blood pressure control,^47^–^50^ those trials were beyond the scope of this review. In middle-aged overweight and obese adults, daily consumption of greater than two and one-half servings of low-fat milk and yogurt for 8 weeks was demonstrated to reduce systolic blood pressure by 2.9 mmHg.^45^ In another study of overweight and obese middle-aged adults, consumption of greater than three and one-half compared with less than three servings of dairy foods, two of which were milk and/or yogurt (fat content not reported), daily for 12 weeks decreased systolic blood pressure by 7.1 ± 3.1 mmHg and diastolic blood pressure by 4.1 ± 1.9 mmHg.^42^ In two studies of borderline hypertensive adults fed just over one serving daily of fermented milk for 8 weeks, no changes in systolic or diastolic blood pressure or components of blood pressure control were observed.^44^,^46^ These studies indicate that at least two and one-half servings of dairy per day is the level at which the effects of dairy on blood pressure are seen. Further studies in normal-weight and obese individuals with and without underlying hypertension will aid in better understanding the relationship between dairy consumption and blood pressure control.

**Table 6:** Effects of dairy and dairy ingredients on blood pressure control: Summary of randomized clinical trials conducted between June 2010 and September 2011, using search terms “dairy,” “milk,” “blood pressure,” and “hypertension”

Reference	Characteristics of participants	Study objective	Dairy servings per day in experimental group	Results
van Meijl & Mensink (2011)^45^	Overweight and obese adults, 50 years of age (*n* = 35)	Investigate the effects of daily consumption of low-fat dairy products on metabolic risk parameters after a period of 8 weeks	>2.5 servings of low-fat milk and yogurt	SBP decreased by 2.9 mmHg (95%CI, −5.5–−0.3; *P* = 0.027), HDL cholesterol decreased by 0.04 mmol/L (95%CI, −0.07–−0.01; *P* = 0.021), apo/A-1 decreased by 0.04 g/L (95%CI, −0.07–−0.01; *P* = 0.016). There was no difference in DBP, total cholesterol, LDL cholesterol, apo B, TAG, NEFA, glucose, insulin, CRP, or plasminogen activator inhibitor-1
Stancliffe et al. (2011)^42^	Overweight and obese adults, 37–40 years of age (*n* = 48)	Determine early and sustained effects of higher dairy consumption versus lower dairy consumption after periods of 7 days, 4 weeks, and 12 weeks	3.5 servings of dairy foods	Consumption of 3.5 servings/day decreased SBP (*P* < 0.01) and DBP (*P* < 0.05), insulin sensitivity (*P* < 0.05), plasma insulin (*P* < 0.05), malondialdehyde (*P* < 0.01), oxidized LDL (*P* < 0.02), TNF-α (*P* < 0.01), MCP-1 (*P* < 0.02), IL-6 (*P* < 0.02), CRP (*P* < 0.02), DBP in only the obese (*P* < 0.05), plasma cholesterol in only the obese (*P* < 0.02), and plasma TAG in only the obese (*P* < 0.05)
Usinger et al. (2010)^46^	Borderline-hypertensive adults, 54–56 years of age (*n* = 94)	Study the effect of *Lactobacillus helveticus* fermented milk on blood pressure control after a period of 8 weeks	>1 serving of fermented milk	No ACE inhibition by fermented milk was detected
Usinger et al. (2010)^44^	Borderline-hypertensive adults, 54–56 years of age (*n* = 94)	Study the effect of *Lactobacillus helveticus* fermented milk on blood pressure control after a period of 8 weeks	>1 serving of fermented milk	No difference in SBP or DBP was detected between lower and higher consumption

*Abbreviations*: apo, apolipoprotein; ACE, angiotensin-converting enzyme; CI, confidence interval; CRP, C-reactive protein; DBP, diastolic blood pressure; HDL, high-density lipoprotein; IL-6, interleukin-6; LDL, low-density lipoprotein; MAP, mean arterial pressure; MCP-1, monocyte chemoattractant protein 1; NEFA, nonesterified fatty acid; sVCAM-1, soluble vascular cell adhesion molecule; SBP, systolic blood pressure; TAG, triglyceride; TNF-α, tumor necrosis factor-α.

## Conclusion

Since the release of the 2010 DGAC report on the 2010 DGA, many studies have been published that make important contributions to the literature on the relationship between dairy consumption, nutrient intakes, and reduced risk of chronic diseases. As the US population continues to underconsume nutrients such as vitamin D, calcium, magnesium, and potassium,^3^,^5^ they also underconsume the recommended daily servings of dairy foods. Since milk and milk products are major contributors of these nutrients to the typical diet in the United States,^8^–^10^ consuming, at minimum, three servings of dairy foods each day would help close the gap on several nutrients that are typically underconsumed.^7^ The totality of the evidence indicates that consumption of three or more servings of dairy per day has beneficial effects on bone in adults, but four servings per day may be necessary to achieve the nutrient status associated with optimal bone health in adulthood. Recent studies continue to build on the evidence that dairy consumption is inversely associated with the development of cardiovascular disease, even at intakes below recommended levels. Milk fat may contribute to the beneficial effects of dairy foods on cardiovascular disease risk, but more research is needed. Recent findings further support the evidence reviewed by the DGAC indicating that meeting recommendations for dairy consumption reduces the risk of type 2 diabetes and has beneficial effects on blood pressure, and that exceeding recommendations may provide further benefits.

In conclusion, meeting and exceeding recommendations for consumption of dairy products each day, in combination with a healthy dietary pattern, leads to better nutrient status, can lead to improved bone health, and is associated with lower blood pressure and a reduced risk of cardiovascular disease and type 2 diabetes.
